# Granulomatosis With Polyangiitis in a Young Lady: Challenges in Achieving Remission

**DOI:** 10.7759/cureus.75139

**Published:** 2024-12-05

**Authors:** Nurul ibtisam Mohammad, Shakiran Gunaseelan, Sangeeta Kuganasan, Noram Mat Saad, Norshamsiah Md Din

**Affiliations:** 1 Ophthalmology, Universiti Kebangsaan Malaysia Medical Centre (UKMMC), Kuala Lumpur, MYS; 2 Ophthalmology, Hospital Tuanku Jaafar, Seremban, MYS

**Keywords:** anca associated vasculitis, anterior scleritis, auto immune optic neuropathy, granulomatosis with polyangiitis (gpa), : immunomodulators, ocular proptosis, rhino-orbital sinusitis

## Abstract

Granulomatosis with polyangiitis (GPA) is a subtype of anti-neutrophil cytoplasmic antibody (ANCA)-associated vasculitis (AAV) that commonly requires aggressive immunosuppression to achieve remission. We present a case of a young Malay lady with recurrent episodes of ANCA-positive nodular anterior scleritis who responded poorly to topical and systemic corticosteroids and relapsed while on methotrexate. A year later, she had epistaxis, and a sino-nasal biopsy confirmed granulomatous vasculitis. While receiving cyclophosphamide, she developed proptosis with optic neuropathy, which resolved with intravenous methylprednisolone. She eventually required rituximab as she was still having relapses on other immunosuppressants. Adequate and targeted treatment with immunomodulators is crucial to achieving disease remission in GPA.

## Introduction

We report a case of a young Malay woman who experienced recurrent episodes of anti-neutrophil cytoplasmic antibody (ANCA)-positive nodular anterior scleritis and who demonstrated a suboptimal response to both topical and systemic corticosteroids. She had a subsequent relapse while undergoing treatment with methotrexate. She presented with epistaxis along with worsening eye complaints. A sino-nasal biopsy was conducted, revealing granulomatous vasculitis, which confirmed the diagnosis of granulomatosis with polyangiitis (GPA). The patient was treated with cyclophosphamide but subsequently developed proptosis and optic neuropathy, necessitating intravenous methylprednisolone. Finally, remission was achieved with rituximab therapy.

GPA is characterized by infiltration of inflammatory cells in vascular tissues leading to necrotizing vasculitis of small and medium-sized vessels [1]. It is common to have both ocular and orbital involvement of varying severity. Approximately a third of patients with GPA have orbital involvement with an average age at presentation of 53.5 years [1]. Vision-threatening complications include exposure to keratopathy, corneal ulceration, corneal perforation, necrotizing scleritis, optic neuropathy, retinal vein occlusion, and exudative retinal detachment [2].

Management of GPA commonly requires adequate immunosuppression with second-line agents to achieve remission [2,3]. With the advent of more precise immunomodulators, targeted immunotherapy may provide remission in recalcitrant cases. We report a case of a young lady with ANCA-positive GPA who presented with ocular features but progressed to involve the orbital structures, which precede systemic involvement despite treatment.

## Case presentation

A 22-year-old Malay lady with no underlying medical illness presented with a one-month history of bilateral eye pain, redness, and reduced vision. The onset of symptoms was asymmetrical, initially affecting the right eye before the left after a week. The eye pain was constant, dull aching, and radiating to the back of the head. There was no reported alopecia, back or joint pain, fever or rashes, nor any constitutional symptoms. She denied any contact with a tuberculosis patient or any high-risk behavior. There was no significant drug history and no family history of malignancy or autoimmune disease.

**Figure 1:**
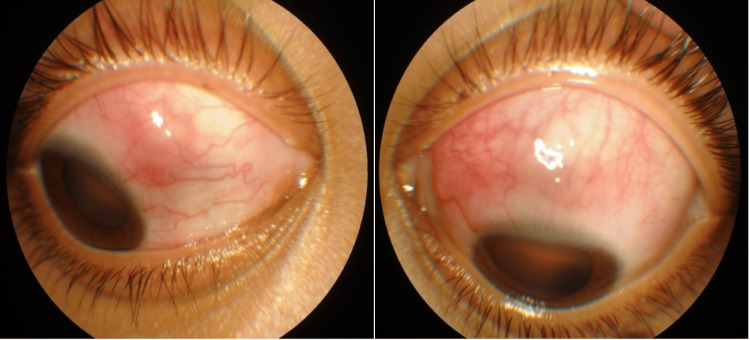
Anterior segment photo of both eyes showing dilatation of deep scleral vessels, hyperemia, and a nodule on the anterior sclera suggestive of nodular anterior scleritis

On examination, the right eye visual acuity (VA) was 6/12, and the left eye was 6/9. There was no relative afferent pupillary defect. Slit lamp examination revealed bilateral sectoral eye redness and dilatation of deep scleral vessels nasally on the right eye and superiorly on the left eye with a scleral nodule suggestive of nodular anterior scleritis (Figure 1). Both the anterior and vitreous chambers were quiet with a normal fundus seen. Systemically, she was overweight with a body mass index (BMI) of 30. She had a saddle nose deformity with bilateral enlarged, non-inflamed tonsils. There was no alopecia, malar rash, oral ulcer, synovitis, digital ulcer, lymphadenopathy, or mass palpable elsewhere on her body.

Initial investigations, including full blood count, renal profile, rheumatoid factor, erythrocyte sedimentation rate, chest X-ray, TB QuantiFERON Gold, hepatitis screening, thyroid profile, lactate dehydrogenase (LDH), and autoimmune investigations including antinuclear antibody (ANA), C3 and C4, extractable nuclear antigen (ENA), anti-proteinase, P-ANCA, and anti-myeloperoxidase, were normal. Anti-proteinase and C-ANCA were, however, positive.

She was diagnosed with bilateral nodular anterior scleritis secondary to ANCA-related vasculitis and was started on oral ibuprofen 200 mg three times a day and topical dexamethasone (0.1%). The response was, however, poor, and oral prednisolone 60 mg daily was commenced, which was tapered to 10 mg daily over three months. She required a maintenance prednisolone dose of 10 mg daily due to frequent recurrences of symptoms. The rheumatology team also co-managed her.

**Figure 2:**
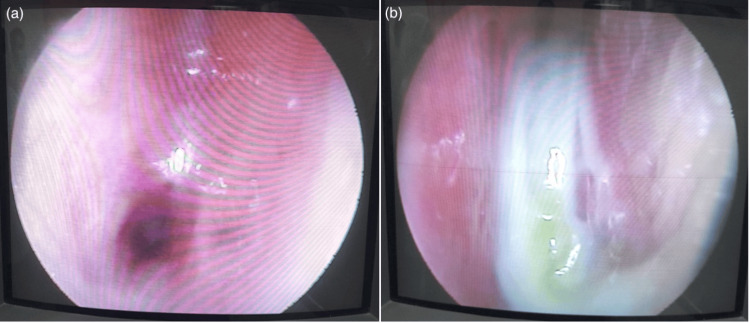
Sino-nasal mucosal biopsy a: thick synechiae between the right inferior turbinate and septum; b: thick mucus, synechiae between the left inferior turbinate and septum

**Figure 3:**
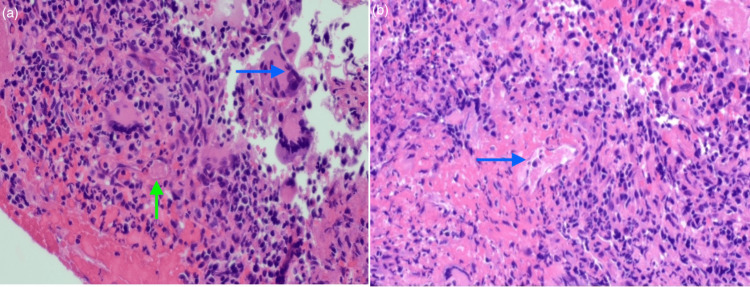
Nasal biopsy a: blue arrow shows poorly formed granulomatous inflammation denoted by the presence of multinucleated giant cells alongside palisading histiocytes, the green arrow points to granuloma formation in a medium-sized artery; b: the arrow indicates vasculitis changes of the medium vessels, the vessel walls were infiltrated by neutrophils with fibrinoid necrosis, and these features are consistent with necrotizing vasculitis with granulomatosis

Despite being on oral steroids, she was still symptomatic, and oral methotrexate was added as a second-line immunosuppressant. After 12 months of the initial symptoms, she developed recurrent epistaxis. A sino-nasal mucosal biopsy (Figures 2, 3) taken by the ear, nose, and throat (ENT) team revealed granulomatous inflammation confirming the diagnosis of granulomatosis with polyangiitis. 

**Figure 4:**
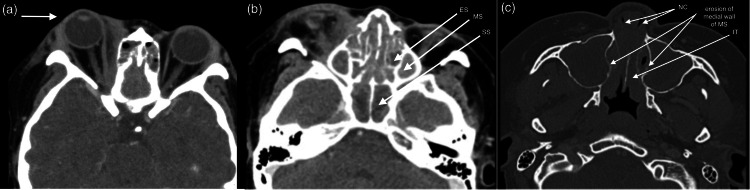
CT scan of the brain and orbit showing right eye proptosis (arrow) a: complete soft tissue opacification was seen in all the paranasal sinuses; b: the medial wall of both maxillary sinuses eroded, and soft tissue thickening was seen in both nasal cavities; c: bilateral middle and inferior nasal turbinate hypertrophied ES: ethmoid sinus; MS: maxillary sinus; SS: sphenoid sinus; NC: nasal cavity; IT: inferior turbinate

Her eye and nasal symptoms improved with methotrexate. However, after 10 months, she developed another episode of scleritis followed by nasal blockage and a vasculitic rash on both lower limbs. Oral methotrexate was discontinued. She was then started on IV cyclophosphamide, of which she completed four cycles. However, during the course of treatment, she developed bilateral eye proptosis with recurrent flare of anterior scleritis, and her vision dropped to 6/18 with grade one relative afferent pupil defect (RAPD) in the right eye. An urgent CT brain, orbit, and paranasal sinuses revealed pansinusitis and nasal polyposis with obliteration of the nasal cavity (Figure 4).

She was given IV methylprednisolone 1 g daily for three doses, followed by a fifth dose of IV cyclophosphamide. Her optic neuropathy and other symptoms resolved, and she responded well to treatment, leading to her discharge.

Unfortunately, the proptosis recurred after two months. We eventually started IV rituximab and added oral azathioprine. She was then able to achieve remission and is currently stable without any symptoms for one year on rituximab and azathioprine.

## Discussion

GPA is the most common subtype of ANCA-related disease, with an incidence of eight to 10 cases per million people [2]. The disease has no gender predilection with a peak incidence in middle age [2]. Two published case reports of GPA in young patients described respiratory tract involvement as the initial presentation of the disease [4,5]. While our young patient had a saddle nose appearance at presentation, she did not have any prior nasal or respiratory symptoms and presented with ocular symptoms first. 

GPA patients can exhibit two different forms, a systemic form with involvement of the kidneys and other organs and a limited form that is confined to the upper and lower respiratory tract [6,7]. Our patient is of limited form with ocular and sino-nasal involvement not involving the kidney or other organs.

The pathogenesis of GPA remains incompletely understood. The characteristic feature of this disease is the concurrent occurrence of vasculitis with granulomatosis. GPA is mainly linked to ANCA antibodies targeting neutrophil proteinase 3 (PR3-ANCA) or myeloperoxidase (MPO) [2,8]. However, it is still unclear how the ANCA antibodies develop. Multiple risk factors have been identified, including but not limited to age, infection, and inflammation in genetically susceptible individuals [2].

Despite its predilection towards the respiratory system, GPA can affect any organ [7,9]. Severe sino-nasal disease may cause thickening of mucosa, subsequent friability, and eventual ulceration. This can present as bloody nasal discharge, recurrent sinusitis, and chronic otitis media [7]. Even in the absence of active disease, one may suffer from symptoms such as dryness, crusting, or even epistaxis due to the loss of lubricating function and mucosal changes [7]. Finally, as seen in our patient, a saddle nose deformity may develop due to damage to nasal cartilage, causing significant cosmetic distress. 

Up to 50% of patients will present with ocular and orbital involvement either due to direct orbital involvement or as a sequelae of disease in the adjacent paranasal sinuses [9]. The signs include dacryoadenitis, orbital myositis, and orbital pseudotumor. Presentation may include, but is not limited to, proptosis, diplopia, sudden onset of pain, or reduced vision [2,10]. Our patient presented with a nodular form of scleritis, which may be potentially blinding. Apart from that, patients can have peripheral ulcerative keratitis, exposure keratopathy, and secondary infection with potential corneal perforation [11].

Diagnosis of GPA is made by a combination of clinical findings and laboratory tests, and confirmation requires a tissue biopsy [9]. The most sensitive test for GPA is the ANCA test, which is both sensitive and specific [9]. In patients with the systemic subtype of GPA, C-ANCA is present 95% of the time, while the remaining cases are associated with positive p-ANCA. Patients with the limited form, however, are only ANCA-positive 60% of the time [9].

Orbital inflammatory disease can lead to compressive optic neuropathy, potentially causing significant vision loss [7]. Vasculitis affecting the arteries that supply the cranial nerves may result in cranial neuropathies and double vision. Ischemic optic neuropathy may lead to sudden and severe vision loss [7]. Rarely, GPA patients can have uveitis, retinal vein occlusion with or without vasculitis, retinitis, chorioretinitis, macular edema, exudative retinal detachment, retinal necrosis, uveal granuloma, vitreous hemorrhage, optic neuritis, and acute multifocal placoid pigment epitheliopathy [2,9]. Our patient developed acute optic neuropathy in the right eye after completing the fourth cycle of IV cyclophosphamide. This is possibly due to inadequate immunosuppression.

Severe forms of the disease are managed with a combination of high-dose corticosteroids and cyclophosphamide until remission is achieved [12]. This may take up to three months, following which maintenance therapy of either azathioprine or methotrexate is maintained for at least two years [12]. In our patient, the methotrexate was stopped after eight months of treatment due to inadequate response to treatment with flares of scleritis and nasal symptoms. The rise of biologics in controlling inflammatory response and regulation of adaptive immunity has given us another avenue in managing cases like this. There have been several studies that have highlighted the benefits of anti-tumor necrosis factor (anti-TNF) in the management of non-infective scleritis secondary to GPA [2,12,13]. Rituximab, in particular, has been found to be a safe and efficacious alternative in managing scleritis, refractory peripheral ulcerative keratitis (PUK), and uveitis associated with GPA [12,13]. The RAVE trial concluded that rituximab was non-inferior to cyclophosphamide in achieving remission for severe ANCA-associated vasculitis and may offer advantages in managing relapsing cases [14]. Our patient eventually required rituximab alongside azathioprine to achieve remission. It currently shows a promising outcome after having no relapses for a year. 

Surgery has limited effectiveness and should not be done in active disease activity. For nasolacrimal duct obstruction, dacryocystorhinostomy may be necessary to create a new outflow pathway and bypass the blockage [9]. For cases with a high risk of ocular perforation, such as necrotizing scleritis or PUK, we may need to consider more invasive treatment like tectonic corneal grafting. Finally, cases with optic nerve compression or severe proptosis due to orbital granuloma may require decompression surgery to restore function [9].

## Conclusions

GPA is a rare disease that can have devastating side effects if not treated properly. Ocular disease may present early and can be the dominant manifestation. Achieving adequate immunosuppression in a young patient can be difficult, with frequent relapses even while on treatment. Our case highlights the challenges in achieving remission with our patient suffering multiple relapses while on treatment. Her relapses often involve different systems necessitating a multidisciplinary approach. Advances in medical therapy have given rise to a multitude of treatment options for patients in the form of biologic agents. We would like to use this case to stress the importance of collaboration between teams and always keeping up to date with the latest available treatment options.
